# Reconstruction algorithm for tunneling ionization with a perturbation for the time-domain observation of an electric-field

**DOI:** 10.1038/s41598-021-92454-y

**Published:** 2021-06-21

**Authors:** Wosik Cho, Jeong-uk Shin, Kyung Taec Kim

**Affiliations:** 1 Center for Relativistic Laser Science, Institute for Basic Science, Gwangju, 61005 Republic of Korea; 2 Department of Physics and Photon Science, Gwangju Institute for Science and Technology, Gwangju, 61005 Republic of Korea

**Keywords:** Optical physics, Optical techniques

## Abstract

We present a reconstruction algorithm developed for the temporal characterization method called tunneling ionization with a perturbation for the time-domain observation of an electric field (TIPTOE). The reconstruction algorithm considers the high-order contribution of an additional laser pulse to ionization, enabling the use of an intense additional laser pulse. Therefore, the signal-to-noise ratio of the TIPTOE measurement is improved by at least one order of magnitude compared to the first-order approximation. In addition, the high-order contribution provides additional information regarding the pulse envelope. The reconstruction algorithm was tested with ionization yields obtained by solving the time-dependent Schrödinger equation. The optimal conditions for accurate reconstruction were analyzed. The reconstruction algorithm was also tested using experimental data obtained using few-cycle laser pulses. The reconstructed pulses obtained under different dispersion conditions exhibited good consistency. These results confirm the validity and accuracy of the reconstruction process.

## Introduction

The temporal characterization of a laser pulse is an essential subject in many laser applications. Many temporal characterization techniques have been developed. Frequency-domain techniques such as autocorrelation^1,2^, frequency-resolved optical gating (FROG)^3^, spectral phase interferometry for direct electric field reconstruction (SPIDER)^4^, dispersion scan (D-scan)^5^, self-referenced spectral interferometry (Wizzler)^6^ and their variations^7,8^ have been widely used thanks to their simple implementation. However, the frequency-domain techniques have several limitations. In general, they work at specific wavelengths in which the nonlinear response of the material can be detected. Implementing these techniques for a laser pulse whose spectrum is broader than that of an octave is difficult. In addition, the temporal range that can be measured is limited by the spectral resolution of the imaging sensor used in the spectrometer. More importantly, they do not directly measure the electric field of the laser pulse. The carrier-envelope-phase (CEP) of the laser pulse cannot be determined. Consequently, a new approach for the complete temporal characterization of a laser pulse for a broad spectral range or a long temporal range have become necessary.

Several time-domain techniques have been developed that overcome the limitations of the frequency-domain techniques. The time-domain technique requires a sub-cycle temporal gate that directly samples a laser field. The streaking technique utilizes attosecond XUV pulses obtained through high harmonic generation as a temporal gate to measure the electric field of a few-cycle laser pulse^9,10^. Similarly, few-cycle laser pulses were used to probe IR and THz pulses^11,12^. In the PHz optical oscilloscope method, a sub-cycle electron motion in a laser field is used as a temporal gate^13^. These techniques can be used for a broad spectral range^14^. Also, the electric field of the laser pulse can be directly measured, including the CEP of the laser pulse. However, these techniques require complicated photoelectron or XUV spectrometers in a vacuum environment, making them very difficult to implement.

A few time-domain approaches that can be implemented in ambient air have recently been developed. A novel approach called tunneling ionization with a perturbation for the time-domain observation of an electric-field (TIPTOE) was demonstrated^15–19^ in which sub-cycle ionization bursts play a role as a temporal gate. Also, there are streaking-like approaches in which electron bunches created by optical field ionization in air^20^ or transition into the conduction band in solids^21,22^ are temporal gates, and a current driven by an orthogonally polarized laser field is measured. These time-domain techniques are highly useful because they can be conveniently implemented in ambient air while they have all the advantages of the time-domain technique. In particular, the TIPTOE technique can be applied using two laser pulses with the same polarization and wavelength. It can be implemented using an inline interferometer, making its implementation very simple. It has been successfully demonstrated for single-cycle^16^, few-cycle^15^, and multi-cycle pulses in the UV, visible, and IR wavelength ranges^17^.

In this study, we demonstrate a reconstruction algorithm developed for the TIPTOE technique. In the TIPTOE method, the amount of ionization is measured using two laser pulses. One laser pulse called “fundamental” is sufficiently intense to induce ionization. The ionization bursts produced by the fundamental pulse play a role of an ultrashort temporal gate. The other laser pulse called “signal” is relatively weak. The signal laser pulse does not induce ionization by itself but interferes with the fundamental pulse and modulates the ionization yield. If the pulse duration of the fundamental pulse is extremely short, ionization may occur only once in a single half-cycle. The modulation of the ionization yield directly represents the electric field of the signal laser field. However, for multi-cycle pulses, ionization occurs in multiple half-cycles. The modulation of the ionization yield becomes slightly longer than the signal laser field in most cases, or it is significantly different from the signal laser field if the fundamental laser pulse is badly chirped. Consequently, a suitable reconstruction process is required to extract the temporal profile of the signal laser pulse from the modulation of the ionization yield.

The temporal profile of the laser pulse has been found by the first-order correction in the previous works^15–19^, which yields accurate results only for limited cases. In this work, we introduce a reconstruction algorithm in which a stochastic gradient-based optimization algorithm is used for an accurate reconstruction. The high-order contributions of the signal laser field to the ionization yield are included in the algorithm, which has two significant advantages. First, the amplitude of the ionization yield modulation is considerably increased to a level much higher than that of noise, significantly improving the signal-to-noise ratio. Second, the higher-order contribution provides additional information on the pulse envelope. Therefore, the TIPTOE measurements become more accurate for a broad dispersion range. With these two critical advantages caused by the new reconstruction algorithm, the TIPTOE method will become a more versatile tool for the temporal characterization of a laser pulse.

## Result

### Normalized ionization yield (NIY)

The temporal profile of a laser pulse was determined using the modulation of the ionization yield in the TIPTOE method. Using the concept of an instantaneous ionization rate that is determined only by the field strength at time $t$ is convenient. The total ionization yield produced in a focal volume $V$ can be obtained by integrating the instantaneous ionization rate $w(\boldsymbol{\rho },t)$ at position $\boldsymbol{\rho }$ as follows:

1 $$N={\int }_{V}^{}{\int }_{t=-\infty }^{+\infty }w\left(\boldsymbol{\rho },t\right)dtdV.$$

Here, we assumed that the ionization rate was sufficiently low so that the depletion of the ground state could be neglected. It should be noted that the instantaneous ionization rate cannot be accurately defined for a single atom. An accurate description of ionization requires a quantum mechanical description in which the amplitude and phase of the transitions and their interference effects are considered. Therefore, the ionization yield of a single atom can be accurately estimated only after the interaction. However, the simplification made using the concept of the instantaneous ionization rate can be justified because the interference effects are averaged out when the ionization yield produced in a focal volume is integrated.

**Figure 1:**
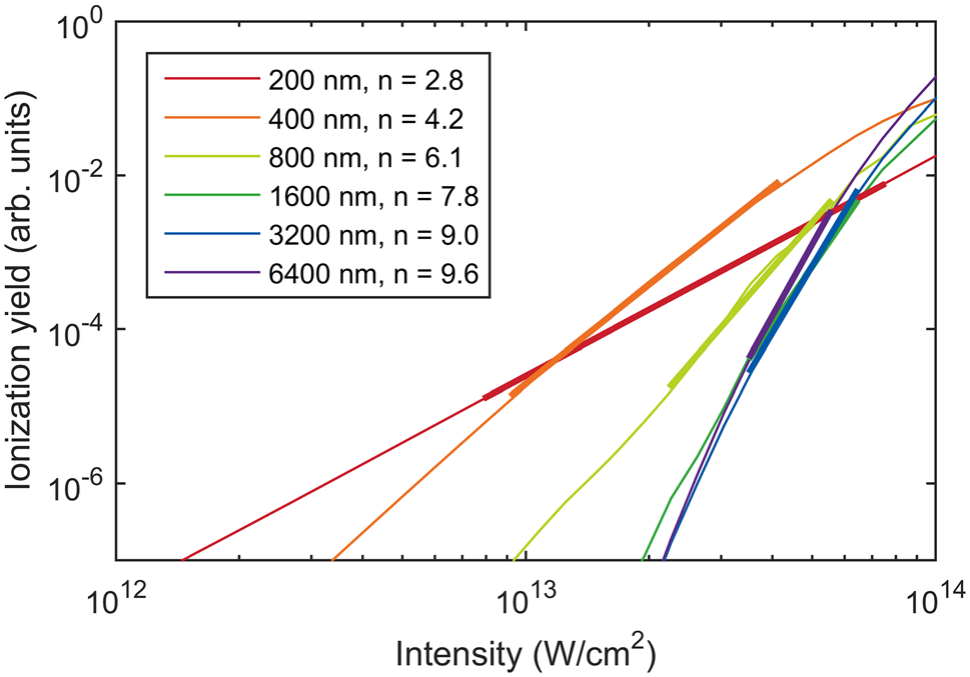
Nonlinear coefficient $n$ calculated using laser pulses at various center wavelengths. Ionization yields (thin solid lines) were calculated using laser pulses with a duration of 3 optical cycles by solving TDSE in 1D at different intensities and wavelengths. The nonlinear coefficient $n$ was obtained by fitting the ionization yields in the range of ${10}^{-2}-{10}^{-5}$ (thick solid lines).

The rate of ionization can be calculated using various models. It should be noted that we used the words ‘tunneling ionization’ in the name of the method TIPTOE to describe the sub-cycle nature of electron wavepackets created by ionization. It does not mean that the fundamental intensity should be high for tunneling ionization. In fact, the fundamental intensity should be kept as low as possible. In this way, the depletion of the ground state can be neglected. The self-phase modulation in an ionizing medium is minimal. Also, the nonlinear coefficient $n$ is high. The choice of the ionization model is not so critical at the low intensity (the intensity range shown with thick lines in Fig. 1 or below). Both multiphoton and tunneling ionization models^23,24^ can be used. For the sake of simplicity, we used the multiphoton ionization model in which the ionization rate is expressed as $w\left(t\right)\propto {I}^{n}={E}^{2n}$. Here, $n$ is the nonlinear coefficient of ionization^25^.

The nonlinear coefficient $n$ can be estimated from the ionization yields obtained as a function of intensity for different wavelengths, as shown in Fig. 1. The nonlinear coefficient corresponds to the slope of the ionization yield in the log–log plot. The slope was estimated for the ionization yield of ${10}^{-2}-{10}^{-5}$ because a reasonably high ionization signal is experimentally measured in ambient air at this ionization level^26^. We found that the reconstruction result was not significantly altered for slightly different $n$ values. Therefore, the nearest integer value obtained for the given center wavelength (i.e., $n=6$ at the center wavelength of 800 nm) can be used even for a broadband laser pulse.

If the TIPTOE method is implemented in different forms, the nonlinear coefficient $n$ should be estimated accordingly. The ionization yield can be measured in different gaseous targets^15^ or from nanostructures^27^. Also, the intensity of fluorescence emission from gas^18^ or solid^28^ can also be measured instead of the amount of the ionization yield. If the nonlinear coefficient is correctly set in these experiments, the reconstruction process can be generally applied.

In the TIPTOE method, an incident laser pulse is split into two laser pulses: the fundamental pulse ${E}_{F}$ and the signal pulse ${E}_{S}$. We assumed that the volume-integrated ionization yield could be approximated by the ionization yield at the focus. We also assumed that the two laser pulses had the same temporal profiles. Then, the ionization yield obtained with time delay $\tau$ can be written as follows:

2 $$N\left(\tau \right)\propto {\int }_{t=-\infty }^{+\infty }{\left[E\left(t-\tau \right)+rE\left(t\right)\right]}^{2n}dt.$$

Here, $r$ is the field strength ratio. The ionization yield $N\left(\tau \right)$ can be directly measured in the experiment. We found that Eq. (2) is computationally expensive. Thus, Eq. (2) can be expanded as follows:

3 $$N\left(\tau \right)={N}^{\left(0\right)}+{N}^{\left(1\right)}+{N}^{\left(2\right)}+{N}^{\left(3\right)}\dots$$

Here,

4 $${N}^{\left(q\right)}={{}_{2n}C}_{q}^{}{r}^{q}{\int }_{t=-\infty }^{+\infty }{\left[E\left(t-\tau \right)\right]}^{2n-q}{\left[E\left(t\right)\right]}^{q}dt$$

The coefficient ${}_{2n}{C}_{q}^{}$ is the binomial coefficient, which is defined as $\left(2n\right)!/\left[\left(2n-q\right)!q!\right]$. It is also useful to define the normalized ionization yield (NIY) as follows:

5 $${\delta }_{N}\left(\tau \right)=\frac{N\left(\tau \right)}{{N}^{\left(0\right)}}-1.$$

The NIY ${\delta }_{N}$ is a dimensionless quantity whose amplitude is determined by the field strength ratio $r$ and the nonlinear coefficient $n$. ${N}^{\left(0\right)}$ is the ionization yield obtained without the signal laser pulse. The maximum value of NIY is obtained when $\tau =0$, that is, $\mathrm{max}\left[{\delta }_{N}\left(\tau \right)\right]={\delta }_{N}\left(\tau =0\right)$. As shown below, the maximum value of NIY is an important parameter to check for accurate TIPTOE measurements.

The two laser beams are superimposed at the focus. If the beam sizes are different, the two beams have different intensity distributions. In addition, the Gouy phase of the two beams varies differently. While the intensity difference between the two laser fields can be safely neglected, the Gouy phase difference would have a noticeable effect because the ionization yield modulations can have slightly different phases at different positions. Integration over the focal volume reduces the amplitude of ionization yield modulation. This volume-averaging effect is more severe for high-frequency components. Therefore, we included the correction factor $V\left(\omega \right)$ in the frequency domain, which is defined as $\mathrm{V}\left(\omega \right)=\mathrm{exp}\left[-{\left(\xi \omega \right)}^{2}/{\omega }_{0}^{2}\right]$. The volume-averaging factor $\xi$ is zero when the sizes of the two laser beams at the focus are identical and increases if the sizes of the two focused laser beams are different. Finally, the NIY can be expressed in the frequency domain as

6 $${\stackrel{\sim }{\delta }}_{N}\left(\omega \right)\propto V\left(\omega \right)\sum _{q=\mathrm{1,2},3\dots }^{}{r}^{q}{}_{2n}{C}_{q}^{}\mathcal{F}{\left\{{\left[E\left(t\right)\right]}^{2n-q}\right\}}^{*}\mathcal{F}\left\{{\left[E\left(t\right)\right]}^{q}\right\}$$

Here, the integration over time is replaced by $\mathcal{F}{\left\{{\left[E\left(t\right)\right]}^{2n-q}\right\}}^{*}\mathcal{F}\left\{{\left[E\left(t\right)\right]}^{q}\right\}$ using the cross-correlation theorem. $\mathcal{F}$ and ${\mathcal{F}}^{*}$ denote the Fourier transform and its complex conjugate, respectively. Equation (6) is an excellent approximation of Eq. (1), which can be estimated efficiently using a fast Fourier transform (FFT) algorithm. We used Eq. (6) in the reconstruction algorithm to calculate a trial NIY.

The CEP of the signal laser pulse can be measured in the TIPTOE measurement only when certain conditions are satisfied. In this work, however, we assumed that the two laser pulses have an identical temporal profile. Therefore, the CEP information of the laser pulse is lost when the Fourier transform and its complex conjugate are multiplied, as shown in Eq. (6). In order to keep the CEP information, the fundamental pulse should be separately prepared so that the phase difference of the two laser pulses can be measured. If the fundamental pulse is a cosine-like pulse, the NIY will directly show the electric field of the signal laser pulse^15^.

### ADAM optimization algorithm

While the NIY is directly measured in a TIPTOE experiment, we want to find the temporal profile of a laser pulse. We could not find an analytical solution to Eq. (6). Therefore, the optimal laser field that minimizes the error between the experimental NIY and trial NIY should be found numerically. This is a typical optimization problem that can be solved numerically.

**Figure 2:**
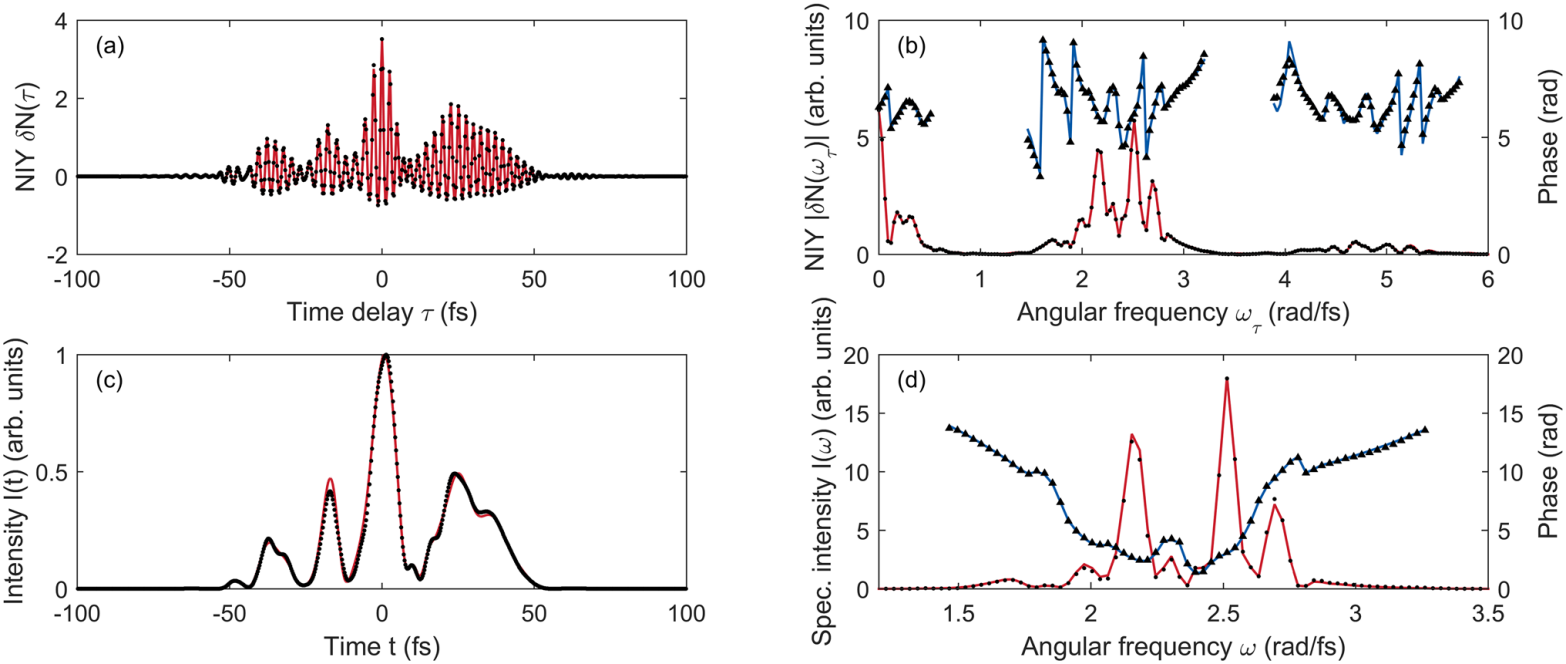
Reconstruction of a laser pulse using ionization yields calculated in a focal volume (3D) by solving TDSE (1D). The peak intensity of the fundamental laser pulse was $2\times{10}^{13}\,\mathrm{W}/\mathrm{cm}^{2}$. The field strength ratio was 0.13. (**a**) Calculated NIY (red lines) and reconstructed NIY (black dots). (**b**) Amplitude (red line) and phase (blue line) of the calculated NIY. Amplitude (black dots) and phase (triangles) of the reconstructed NIY. (**c**) Intensity of the original laser pulse (red line) and the reconstructed laser pulse (black dots). (**d**) Spectral intensity (red line) and phase (blue line) of the original laser pulse. Spectral intensity (black dots) and phase (triangles) of the reconstructed laser pulse.

Many optimization algorithms are available depending on the problems to be solved. One aspect of the TIPTOE optimization problem is that it has many fitting parameters. The spectral amplitude and phases in the spectral range of interest are the fitting parameters to be determined. The field strength ratio $r$ and the volume-averaging factor $\xi$ are also fitting parameters. For ${N}_{Fit}$ spectral amplitudes and phases in the spectral range of interest, there are $\left({N}_{Fit}+2\right)$ fitting parameters. For the data shown in Fig. 2, ${N}_{Fit}\hspace{0.17em}$= 146 for the spectral range from $1.3$ to $3.5\times {10}^{15}\mathrm{r}\mathrm{a}\mathrm{d}/\mathrm{f}\mathrm{s}$ with a temporal range of 200 fs. If the temporal range of the data is very long, ${N}_{Fit}$ can be very large. The other aspect of the TIPTOE optimization problem is that a good initial guess is available unless the fundamental laser pulse is badly chirped. NIY is an excellent approximation of the optimal laser field. In most cases, the signal laser field is slightly shorter than the ionization yield modulation. Considering these aspects, we chose a first-order gradient descent algorithm, which works efficiently even with many fitting parameters when a reasonably good initial guess is provided.

The cost function that needs to be minimized is the root-mean-square (RMS) error, which is defined as follows:

7 $${\mathrm{\varepsilon }}_{\mathrm{R}\mathrm{M}\mathrm{S}}=\frac{1}{{N}_{\omega }}\sqrt{{\sum }_{\omega }^{}{\left|{\stackrel{\sim }{\delta }}_{N}^{EXP}\left(\omega \right)-{\stackrel{\sim }{\delta }}_{N}^{REC}\left(\omega \right)\right|}^{2}}$$

Here, ${N}_{\omega }$ is the number of data points. In the gradient descent algorithm, the reconstructed NIY (${\stackrel{\sim }{\delta }}_{N}^{REC}$) is calculated using a trial laser field. An initial guess for the trial laser field is obtained using the measured NIY (${\stackrel{\sim }{\delta }}_{N}^{EXP}$). The gradient of the error $\partial {\varepsilon }_{RMS}/\partial {p}_{i}$ for the ${i}^{\mathrm{t}\mathrm{h}}$ fitting parameter ${p}_{i}$ is calculated for each fitting parameter using the trial solution. Then, a new trial solution that reduces the error is obtained by adding $-\mathrm{\alpha }\partial {\varepsilon }_{RMS}/\partial {p}_{i}$ to ${p}_{i}$, where $\mathrm{\alpha }$ is the step size. This process is repeated until the stop condition is satisfied.

There are several gradient descent algorithms, depending on how the step size is determined during iterations. We chose the ADAM algorithm, which performs well in many applications^29^. The name ADAM is derived from adaptive moment estimation. In the ADAM algorithm, the step size $\mathrm{\alpha }$ is updated using the exponential moving averages of the gradients over the iterations. The hyper-parameters ${\beta }_{1}$ and ${\beta }_{2}$ used to calculate the momentum of the update were fixed to values of 0.9 and 0.999, and the initial step size was 0.01, as recommended in the literature^29^. The iteration stopped if the number of iterations reached 2000, or the standard deviation of the RMS error for the last 50 iterations was below ${10}^{-7}$.

### Reconstruction of theoretically calculated NIYs

**Figure 3:**
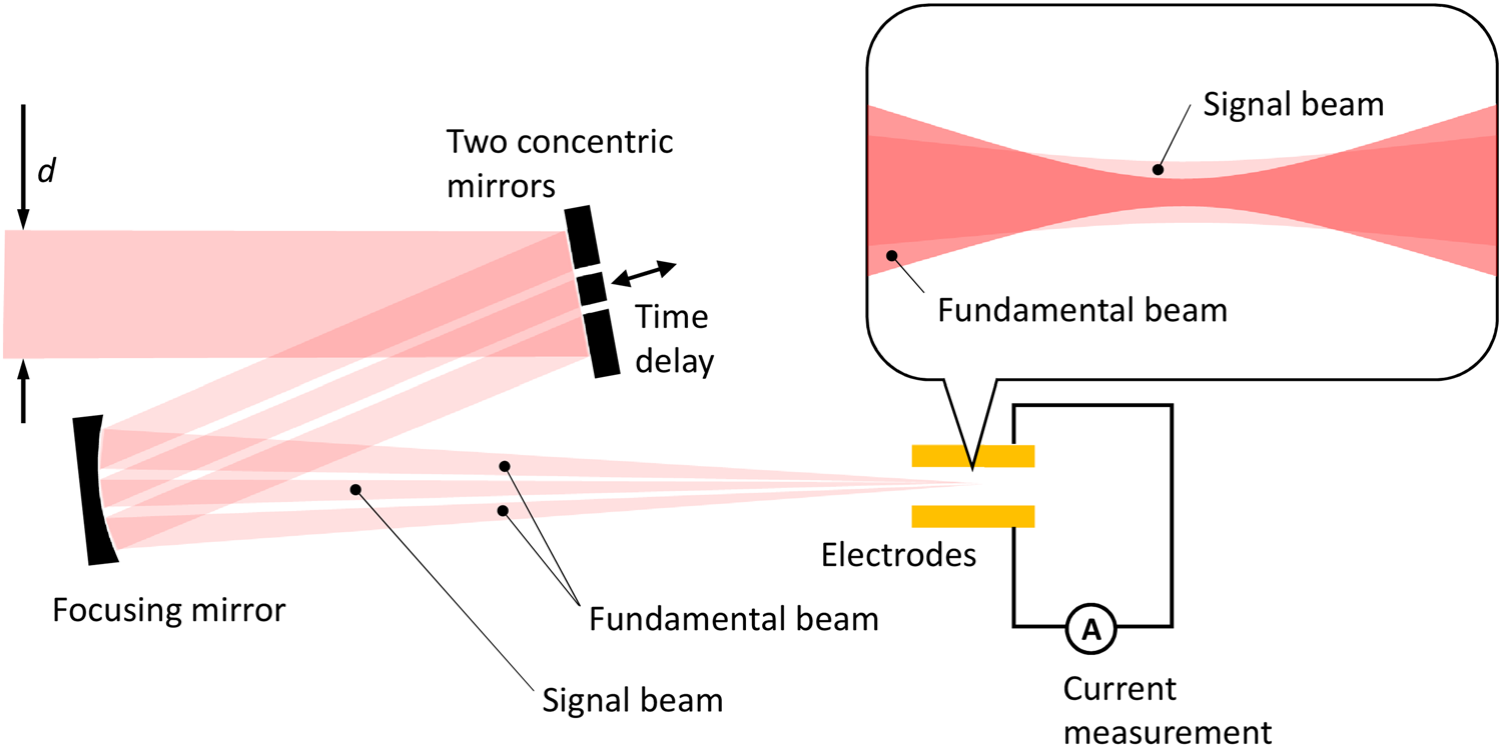
Experimental setup for the TIPTOE measurement. An incident laser beam with a diameter of $d$ was reflected using two concentric mirrors. Both beams were focused using a focusing mirror. The outer beam is the fundamental beam that makes a relatively small and intense beam at the focus. The inner beam is the signal beam that makes a relatively big and weak beam at the focus. The ionization yield was measured using the transimpedance amplifier attached to the metal electrodes.

The validity of the reconstruction process was tested using the ionization yields calculated numerically. To consider a realistic condition used in the experiments and to justify the assumptions made to derive Eq. (6), we assumed conditions similar to those used in the experiment. We assumed that the two laser beams were prepared using annular and small inner mirrors, as in the experimental setup shown in Fig. 3. The signal laser beam was reflected from the inner mirror, whose diameter was fixed at 2 mm. The fundamental laser beam was reflected using an annular mirror with an inner diameter of 2.5 mm. The outer diameter of the incident laser beam $d$ was varied such that the field strength ratio $r$ at the focus could be adjusted. The intensity of the incident laser beam was set such that the peak intensity of the fundamental laser beam was $2\times {10}^{13}\,\mathrm{W}/\mathrm{cm}^{2}$ at the focus. The propagation of the two beams was calculated using the Huygens-Fresnel principle. Thus, the two laser fields were obtained as functions of position and time in the focal volume.

The ionization yield was calculated by solving the time-dependent Schrodinger equation in 1D using a softcore potential defined as $\mathrm{V}\left(x\right)=1/\sqrt{{x}^{2}+{a}_{2}}$. The constant ${\mathrm{a}}_{2}$ was set to 1.72 to obtain an ionization rate similar to that obtained in the experiment^26^. The ionization yields obtained at different positions were integrated as a function of time delay. NIY was calculated using ${\mathrm{\delta }}_{\mathrm{N}}\left(\tau \right)=N\left(\tau \right)/{N}^{(0)}$. Here, ${N}^{(0)}$ is the ionization yield obtained without the signal laser pulse. In this work, we estimated ${N}^{(0)}$ by the ionization yield obtained at a large time delay, where the two laser pulses do not temporally overlap.

In the first example, we calculated the normalized ionization yield using a complicated laser pulse, as shown in Fig. 2. The laser pulse was made by adding a few arbitrarily chirped pulses, as shown in Fig. 2(c). In this calculation, the ionization yield was calculated in a focal volume in 3D as a function of time delay. The iris size was set to 6 mm. The field strength ratio $r$ was 0.13. Because this is a considerably high ratio compared to the value used in previous works^15–17^, the NIY exhibited an asymmetric modulation, as shown in Fig. 2(a). The positive side of NIY was much higher than that of the negative side of NIY. The maximum NIY was 3.5. This implies that high-order modulations contribute significantly.

The spectrum of NIY, $\delta N\left(\omega \right)$, also clearly shows high-order contributions, as shown in Fig. 2(b). The spectrum of the NIY between 1.5 to 3.5 rad/fs is mainly contributed by ${N}^{(1)}$ that contains $E$ with a small contribution of ${N}^{(3)}$ that contains ${E}^{3}$ as in Eq. (3). The spectrums near dc (0 – 1 rad/fs) and from 3.5 to 6 rad/fs are attributed to ${N}^{(2)}$ and ${N}^{(4)}$, which contain ${E}^{2}$ and ${E}^{4}$. Notably, the NIY near DC is attributed to the DC component of ${E}^{2}$. The DC component in the spectrum of the NIY does not contain phase information, but it contains information on the pulse envelope, which cannot be found in the first-order approximation used in previous works^15–17^.

Reconstruction was performed using ADAM. It was completed after 170 iterations, which took approximately 3 s using an ordinary personnel computer (Intel i7-7700 CPU, 8 GB RAM, Windows 10). The number of data points for FFT $\left({N}_{FFT}\right)$ was 768. We found that the calculation time was proportional to $\sim {N}_{FFT}^{2}$. The reconstructed NIY agreed well in both the time and frequency domains, as shown in Fig. 2(a,b). The intensity profile and the spectrum of the reconstructed laser pulse also show excellent agreement with that of the original laser pulse, as shown in Fig. 2(c,d). These reconstruction results support the validity of the reconstruction.

### Optimal experimental parameters for an accurate TIPTOE measurement

The TIPTOE measurement is accurate when the experimental parameters such as the field strength ratio $r$ and pulse duration are properly set. We performed a series of calculations to test a reliable range of the field strength ratio and pulse duration. We used a chirped Gaussian pulse with a transform-limited duration of 3.5 fs with a central wavelength of 800 nm. Because the calculation of the NIYs in a 3D focal volume is very time-consuming, we calculated NIYs along the beam axis only for this analysis. The field strength ratio $r$ was set by changing the iris diameter $d$. As the field strength ratio changes, the maximum values of NIY, $\mathrm{max}\left({\delta }_{N}\right)$ or ${\delta }_{N}\left(\tau =0\right)$, also change. The pulse duration was varied by imposing a group delay dispersion (GDD) on the laser pulse.

**Figure 4:**
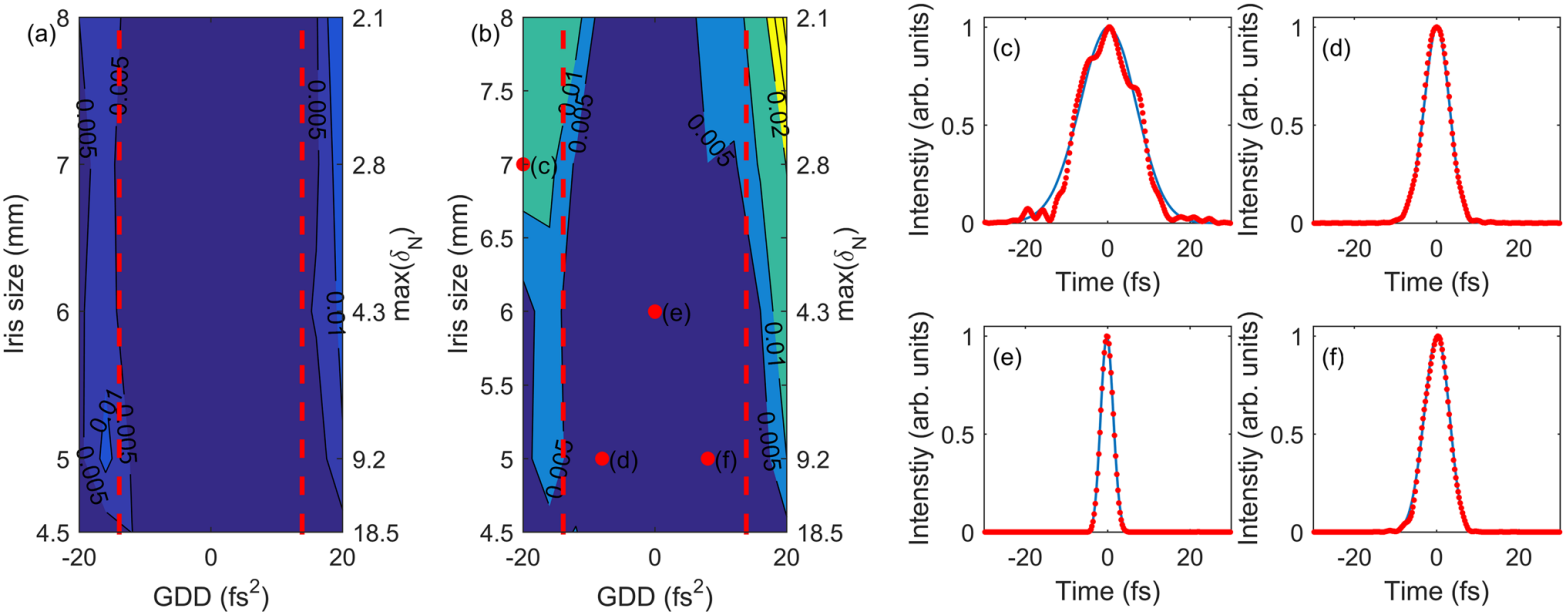
Reconstruction results obtained using chirped Gaussian pulses for different maximum NIYs and GDDs. (**a**) Normalized differences obtained using Gaussian pulse without noise. (**b**) Normalized differences with 1% NRMS power fluctuation of a laser pulse. The duration of the laser pulse was 3.3 ${\mathrm{\tau }}_{\mathrm{T}\mathrm{L}}$ at a GDD of $\pm 13.9\,{\mathrm{f}\mathrm{s}}^{2}$, as shown with red dashed lines in (**a**) and (**b**). (**c**–**f**) Intensity profiles of the original laser pulse (blue line) and reconstructed intensity profiles (red dots) obtained at the conditions denoted with red circles in (**b**).

To estimate the accuracy of the reconstruction in many different cases, we defined the normalized difference that quantifies the difference between the original intensity ${I}^{org}\left(t\right)$ and the reconstructed intensity ${I}^{rec}(t)$ by $\int {\left({I}^{rec}\left(t\right)-{I}^{org}\left(t\right)\right)}^{2}/\int {\left({I}^{org}\left(t\right)\right)}^{2}$. We found that the two pulses were identical when the normalized difference was less than 0.005. The normalized differences obtained for different iris sizes and GDDs are summarized in Fig. 4(a). The accuracy of the reconstruction does not depend significantly on the iris size (or field strength ratio $r$) in this case. However, the reconstruction results are more accurate when the GDD is lower than 13.9 ${\mathrm{f}\mathrm{s}}^{2}$. Within this range, the pulse duration is shorter than approximately $3.3{\mathrm{\tau }}_{\mathrm{T}\mathrm{L}}$. Here, ${\mathrm{\tau }}_{\mathrm{T}\mathrm{L}}$ is the transform-limited duration, which means that the TIPTOE measurement is accurate when the duration of the laser pulse is shorter than $3.3{\mathrm{\tau }}_{\mathrm{T}\mathrm{L}}$.

The restriction on the pulse duration of the TIPTOE measurement can be explained using Eq. (6). NIY is the sum of cross-correlations. The first order, which can be written as ${N}^{\left(1\right)}\left(\omega \right)\propto G\left(\omega \right)E\left(\omega \right)$, contributes to the NIY comes the most significantly. Here, $G\left(\omega \right)=\mathcal{F}{\left\{{\left[E\left(t\right)\right]}^{2n-1}\right\}}^{*}$. The reconstruction process presented here is equivalent to finding a solution of $N(\omega )/G\left(\omega \right)$. When the pulse duration is long, the bandwidth of $G\left(\omega \right)$ becomes narrower, and its value can be extremely small at both sides of the spectrum, causing a zero-divide-by-zero problem. Therefore, the pulse duration should not be too long compared to the transform-limited duration. When the pulse duration is $\left(\sqrt{2n-1}\right){\tau }_{TL}$, the bandwidth of $G\left(\omega \right)$ is the same as the bandwidth of $E\left(\omega \right)$. If the pulse duration is longer than $\left(\sqrt{2n-1}\right){\tau }_{TL}$, the reconstruction can be inaccurate. Therefore, the duration limit that yields accurate reconstruction results is approximately $3.3{\mathrm{\tau }}_{\mathrm{T}\mathrm{L}}$ when $\mathrm{n}=6$ at 800 nm. The duration limit is slightly low for short wavelengths ($2.6{\mathrm{\tau }}_{\mathrm{T}\mathrm{L}}$ when $n=4$ at 400 nm) and high for long central wavelengths (for example, $4.1{\mathrm{\tau }}_{\mathrm{T}\mathrm{L}}$ when $n=9$ at 3200 nm). The TIPTOE measurement is reliable when the pulse duration is shorter than this duration limit.

Note that the duration limit applies only to the fundamental laser pulse that determines the bandwidth of the $G\left(\omega \right)$. We assumed that the two laser pulses were identical in this study. Therefore, the reconstruction is accurate within the duration limit. To measure a badly chirped signal laser pulse whose duration is extremely long, the two pulses can be completely separated, and a near-chirp-free fundamental laser pulse can be used to measure the signal pulse. In such a case, the ionization yield modulation is very similar to the temporal shape of the signal laser pulse. An arbitrarily chirped signal pulse can be measured without a duration limit.

The accuracy of the reconstruction is also affected by noise. We performed the same calculation with noise, as shown in Fig. 4(b). We assumed that the power fluctuation (normalized root-mean-square (NRMS)) of the laser pulse was approximately 1%. The calculated NIY showed an NRMS noise of approximately 6%. The normalized difference of the reconstruction result was analyzed in the same way as in Fig. 4(a). The reconstruction results depended both on the maximum NIY and GDDs, as shown in Fig. 4(b). When the maximum NIY was smaller than 3, the reconstruction was accurate for a narrow GDD range (or duration). In contrast, the reconstruction was still accurate for the duration range of $3.3{\mathrm{\tau }}_{\mathrm{T}\mathrm{L}}$ when the maximum NIY was greater than 3. This was natural because the signal-to-noise ratio improved when the maximum NIY was large.

The reconstructed pulses obtained under different conditions, as marked in Fig. 4(b), are shown in Fig. 4(c–f). They showed good agreement when the maximum NIY was large and their pulse duration was shorter than $3.3{\mathrm{\tau }}_{\mathrm{T}\mathrm{L}}$, as shown in Fig. 4(d–f). If not, the reconstruction result showed a significant deviation from the original pulse, as shown in Fig. 4(c). However, even in this case, the pulse duration (or overall temporal profile) was still reasonably accurate. Based on these calculations, we can conclude that an accurate reconstruction is achieved when the maximum NIY is high within the duration limit. In real experiments, the maximum NIY cannot be infinitely high because of the dynamic range of the current measurement device with a reasonably high ${N}^{\left(0\right)}$. The maximum value of the maximum NIY that could be obtained in an experiment was approximately 20. Consequently, a maximum NIY of approximately 3 – 20 is a good range for accurate reconstruction.

In most practical applications, the pulse duration is controlled by adjusting the amount of dispersion using a grating pair or material dispersion. Because ionization is highly nonlinear to the intensity of a laser pulse, the condition for the shortest possible duration can be easily determined by adjusting the amount of dispersion to obtain the maximum ionization yield. This condition is a good starting point for an accurate reconstruction. The shortest pulse can be measured near this dispersion condition.

In addition, the experimental data always contain a certain amount of noise. The easiest way to reduce the noise is to increase the number of data points or to average multiple NIYs. We found that it is more effective to average multiple NIYs than to increase the number of data points because the slowly varying power fluctuation affect the reconstruction rather than shot-to-shot noise. If the power fluctuation of the laser system is too high, a differential measurement can be implemented in which ${N}^{\left(0\right)}$ is separately measured in an additional electrode^17^. In principle, differential measurements can completely suppress the noise caused by power fluctuations. Therefore, the experimental conditions for accurate TIPTOE measurements can be achieved in most practical applications.

### Reconstruction of experimental results

**Figure 5:**
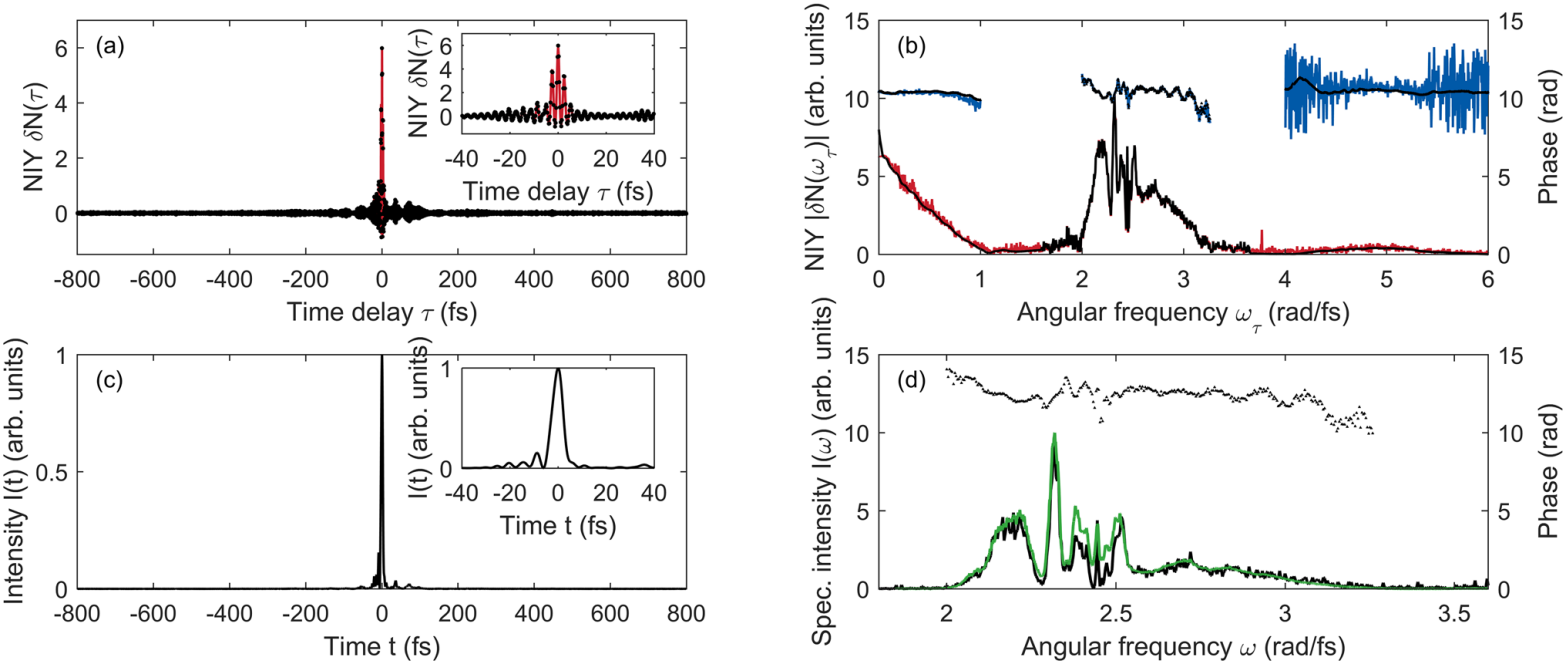
Reconstruction of few-cycle laser pulses obtained in an experiment. (**a**) The experimental NIY (red line) and the reconstructed NIY (black dots) in the time domain. The same plot for a short range of the time delay is shown in the inset. (**b**) The spectral amplitude (red line) and phase (blue line) of the experimental NIY and that (black dots and triangles) of the reconstructed NIY. (**c**) The reconstructed intensity profile in the time domain. The same plot for a short range of the time delay is shown in the inset. (**d**) The spectral intensities (black line) and phases (triangles) of the reconstructed spectrum. The spectral intensity of the spectrum obtained using a spectrometer (green).

The reconstruction algorithm was also tested using experimental data with few-cycle laser pulses; the experimental setup is shown in Fig. 3. The amount of dispersion of the pulse was controlled by a pair of BK-7 glass wedges (see Methods). The NIYs were measured five times and averaged, as shown in Fig. 5(a). The iris diameter of the device was controlled to achieve a reasonably high maximum NIY. The maximum NIY was 6, as shown in Fig. 5(a). The reconstruction was performed using NIY, as shown in Fig. 5(a). The NIYs were reproduced in both the time and frequency domains, as shown in Fig. 5(a,b). The pulse duration of the laser was 5.1 fs, as shown in Fig. 5(c).

The spectral amplitudes and phases are presented in Fig. 5(d). The reconstructed spectrum showed good agreement with the spectrum independently measured using a spectrometer, supporting the validity of the measurement. The resemblance between the reconstructed spectrum and the spectrum measured by the spectrometer was remarkable, considering that the reconstructed spectrum was obtained using the NIY measured in the time domain. This means that TIPTOE can also be used to measure an accurate spectrum. The spectral resolution of the measurement was approximately 1 nm at 800 nm, which can be improved by measuring the NIY over a longer temporal range of the measurement. This capability will be very useful in MID IR wavelengths, at which it is difficult to find a spectrometer with a good spectral resolution over a broad spectral range.

**Figure 6:**
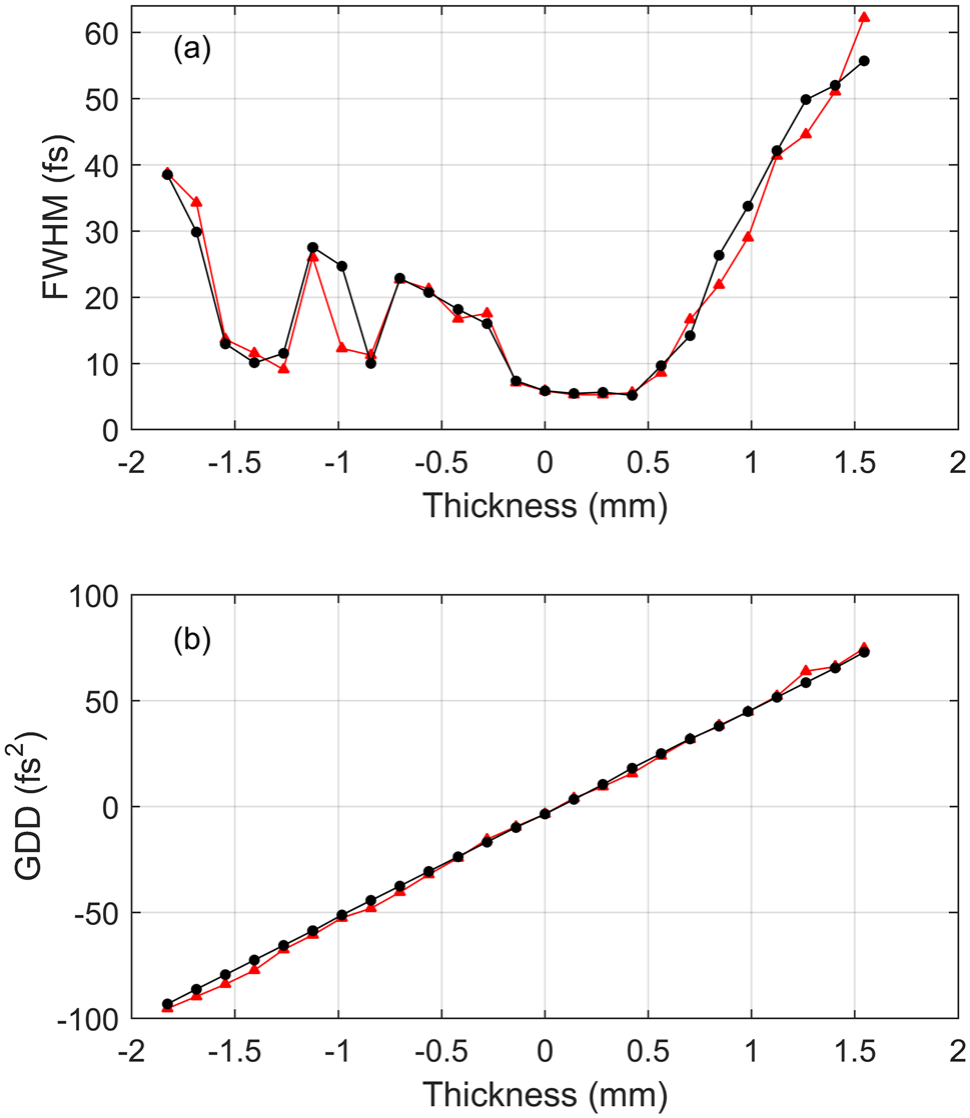
Consistency test of reconstruction of the chirped laser pulses. The laser pulses were measured at different dispersion conditions. The amount of dispersion was controlled by a BK7 glass wedge. (**a**) The reconstructed pulse durations of the chirped laser pulses obtained in experiments (red triangles) and the pulse durations of artificially chirped pulses. The artificially chirped pulses were calculated by adding a theoretical dispersion on the reconstructed laser pulse obtained at the minimum GDD condition. (**b**) The reconstructed GDD of the chirped laser pulses (red triangles) and the GDD of the artificially chirped pulse (black circles).

The NIYs were also obtained under different dispersion conditions by changing the thickness of the BK-7 glass wedge. Reconstruction was performed independently on each dataset. We then checked whether the reconstruction results were consistent. The pulse durations and GDDs were obtained from the reconstructed laser pulse, as shown in Fig. 6(a,b). Then, we calculated an artificially chirped pulse using the reconstructed pulse obtained at the minimum GDD condition. The pulse durations and GDDs of these artificially chirped pulses are also shown in Fig. 6(a,b). They show good agreement, confirming the validity and accuracy of the TIPTOE measurements and their reconstruction.

## Summary

We developed a reconstruction algorithm for the TIPTOE method. We tested the reconstruction process under various conditions. The reconstruction results were analyzed to determine the optimal conditions for accurate reconstruction. Because the high-order contribution of the signal laser pulse was included in the reconstruction algorithm, the signal-to-noise ratio was significantly improved.

The accuracy of the reconstruction algorithm was analyzed using different parameters such as the field strength ratio and pulse duration. The reconstruction is accurate when the maximum NIY is large (from 3 to 20) within a certain duration limit (approximately $3.3{\mathrm{\tau }}_{\mathrm{T}\mathrm{L}}$ at 800 nm). The reconstructed pulses obtained under different dispersion conditions exhibited very good consistency. These results confirmed the validity and accuracy of the reconstruction process. With the new reconstruction algorithm, the TIPTOE method will become a more useful tool for the temporal characterization of a laser pulse.

## Methods

We used a titanium sapphire laser (Femtolasers, Femtopower X CEP4) that generates 800-nm 30-fs laser pulses at a repetition rate of 1 kHz. The output laser pulse was coupled with a stretched hollow-core fiber and a set of chirped mirrors for pulse compression (Ultrafast innovation, PC37_4). The power fluctuation of the few-cycle output was approximately 1%. The TIPTOE device (SourceLAB, TIPTOE) used in the experiment had two electrodes, but we used only one electrode, as shown in Fig. 3. In this device, the cable noise is almost entirely suppressed by the transimpedance amplifier directly attached to the electrode, enabling accurate measurement of the ionization yields. We adjusted the laser pulse energy to obtain a reasonably high voltage (0.2 V) from the transimpedance amplifier attached to the electrode when the two pulses did not overlap. The pulse energy used for the TIPTOE measurement was approximately 1 $\mathrm{u}\mathrm{J}$.
